# It’s the AI’s fault, not mine: Mind perception increases blame attribution to AI

**DOI:** 10.1371/journal.pone.0314559

**Published:** 2024-12-18

**Authors:** Minjoo Joo

**Affiliations:** Department of Social Psychology, Sookmyung Women’s University, Seoul, Korea; Marquette University College of Education, UNITED STATES OF AMERICA

## Abstract

Can artificial intelligences (AIs) be held accountable for moral transgressions? Current research examines how attributing human mind to AI influences the blame assignment to both the AI and the humans involved in real-world moral transgressions. We hypothesized that perceiving AI as having a human mind-like qualities would increase moral blame directed towards AI while decreasing blame attribution to human agents involved. Through three empirical studies—utilizing correlational methods with real-life inspired scenarios in Study 1 and employing experimental manipulations in Studies 2 and 3—our findings demonstrate that perceiving mind in AI increases the likelihood of blaming AIs for moral transgressions. We also explore whether it also diminishes the perceived culpability of human stakeholders, particularly the involved company. Our findings highlight the significance of AI mind perception as a key determinant in increasing blame attribution towards AI in instances of moral transgressions. Additionally, our research sheds light on the phenomenon of moral scapegoating, cautioning against the potential misuse of AI as a scapegoat for moral transgressions. These results emphasize the imperative of further investigating blame attribution assigned to AI entities.

## Introduction

Amidst the COVID-19 pandemic in 2020, resident physicians at Stanford Medical Center, who were actively engaged on the front lines, discovered that they were not included in the prioritized distribution of vaccine doses. Out of over 1,300 resident physicians, only 7 received doses, while certain administrators and remote workers had made the list. At least 100 residents protested. Leadership apologized, attributing the oversight to the “very complex algorithms.” Some protesters held signs stating, “We don’t trust them,” while chanting, “Algorithm sucks” [1].

This incident exemplifies the challenge of assigning responsibility when autonomous machines lead to moral consequences, illustrating what is often referred to as the responsibility gap [2]. This gap can create opportunities for individuals overseeing such technologies to avoid their responsibilities. By placing blame on artificial intelligence (AI) for moral violations, those in charge may deflect accountability onto AI, using it as a scapegoat. Moreover, anthropomorphizing of AI can make it worse. The language used by protestors aligns with the tendency in recent media to humanize artificial intelligence (AI) software, labeling them as spreading hate, being sexist, and being racist. Because intentionality and mental state is the important precursor of blame attribution [3], attributing human mind to AIs could make it easier to place blame and responsibility to them.

Considerable scholarly attention has been paid to responsibility gap caused by accident involving autonomous vehicles (AVs), or moral dilemmas in AI decision-making [4–8]. However, comparatively little attention has been given to explicit moral transgressions instigated by AIs. In the current research, we use real-life instances of moral violations caused by AI and empirically investigate the assignment of moral blame and responsibility to AI. In particular, we identified attribution of human-like mental faculties (i.e., mind perception) to AI as a critical factor in assigning blame to AI. Additionally, we explored the possibility of scapegoating, wherein blame shifts away from the individuals involved and stakeholders (e.g., programmers, companies, governments).

### AI and blame attribution

Can AI be held morally responsible? According to previous studies, individuals are inclined to blame AI for various transgressions, such as causing environmental damage [9], choosing to hit an innocent pedestrian [10], and making decisions that cause medical accidents and military harms [11]. As AIs are not social entities, this raises intriguing questions about moral blame attribution. Blame can arise from cognitive evaluations about whether the transgressor could have acted differently, whether they foresaw the consequences, and whether they violated norms intentionally [12–14]. In this study, blame is defined as the ascription of responsibility for the transgression, with the cognitive process being treated as the central component of blame. Blame attribution holds significant social importance because it involves pointing a finger at a person as responsible. The person being blamed is likely to bear the costs of punishment and criticism, while the one assigning the blame carries the risk of wrongfully accusing an innocent individual [3]. Since AIs do not carry the same risks and costs associated with being blamed, they may become convenient targets. For instance, it has been shown that individuals tend to blame AIs when it serves their self-interest bias [10].

Not all AIs are treated as moral agents who can be held responsible. Models of moral agency suggest that only entities possessing agentic mental capacities—such as awareness, the ability to think, and the capacity to plan—could be considered morally responsible [15]. Additionally, individuals tend to make systematic judgments about blame, considering factors such as the agent’s intentionality and the quality of moral reasoning, given the high social risks associated with blame attribution [3]. Taken together, it appears crucial for AIs to be perceived as having mental capacities capable of making moral judgments to be considered moral agents who can be blamed. Indeed, previous studies have shown that when individuals perceive greater agentic capacities in AIs, they are more likely to assign higher blame to them [16,17] and are more inclined to punish them for their actions [18].

### Mind perception in AI and moral scapegoating

Perceiving AIs as mentally capable can result from engaging in the process of inferring minds in non-human entities, a phenomenon known as anthropomorphism [19]. When individuals perceive minds in AI, they not only see it as having mental capacities for rational thoughts (agency) but also as being capable of conscious feelings (experience; [20], which are distinctively human characteristics. While many previous studies have emphasized the perception of agency as an important precursor to AIs having moral responsibilities, the perception of experience is also crucial. Social-emotional abilities, such as understanding and valuing other people’s feelings, are fundamental to comprehending the ramifications of behaviors and judgments. These abilities enable individuals to truly grasp the impact of their actions on others. Further, because individuals tend to perceive AI as capable of some degree of agentic capabilities but devoid of experiential capabilities, increasing the perception of the latter seems crucial in AI mind perception [20]. Indeed, Young & Monroe (2019) found that perceiving the experiential capacities of AI was related to increased trust in decisions made by AI in moral dilemma situations, indicating perception of experience enhances the view of AIs as moral agents.

Hence, mind perception of AIs is a deciding factor of whether blame attribution would be easily made for AIs in the face of the moral transgression. Previous studies have shown that anthropomorphism can change attribution process of blame by altering the appearance and behavior of robots. For example, autonomously behaving AIs were more likely to be blamed or take credit than non-autonomous AIs [21,22]. More importantly, Shank et al., [23] measured mind perception in the context of AI moral transgression to examine the link between mind perception of AI and blame. They found that perceived mind in AI was linked to higher blame in AI. However, they did not manipulate the mind perception, and only included mind-perception as a covariate along with other manipulations, obscuring the clear causal path between mind perception and blame attribution.

Additionally, increase in blame directed towards AI can indicate a decrease in blame attributed to other agents, potentially facilitating scapegoating. Team decision-making is inherently susceptible to moral scapegoating due to the dynamics of shared responsibility [24,25], and this phenomenon may extend to decisions involving AIs. However, the findings from the literature are varied. Some studies indicate that the perception of autonomy in vehicles is associated with heightened blame directed towards some stakeholders (e.g., manufacturers, government entities) following an accident [4,26]. In contrast, some suggest that AIs can share blame in joint decision-making setting. For instance, when both human and AI were agents, individuals tend to attribute responsibility to self when the outcome was positive, but the reverse pattern emerged for the negative outcomes [10]. This diverging pattern can be explained by the degree to which AIs are perceived as having minds. When individuals perceive AIs as possessing minds, they may be viewed as adequate sharers of blame. However, if AIs are perceived as lacking minds, the tendency for moral scapegoating may not manifest, as other agents are perceived to be in control of and responsible for the actions of the AI.

### The present study

The current research serves three primary objectives. Firstly, we aim to broaden understanding of the blame directed towards AI in real-world moral transgression contexts. Despite instances where AIs are implicated in overt moral violations, such as exhibiting racial biases or displaying aggression, existing studies have predominantly focused on moral dilemmas involving AIs [27]. Explicit moral faults involve clear-cut instances of AI behavior that contravene widely accepted moral norms or standards thereby enabling straightforward blame attribution. Second, we sought to replicate and expand upon the relationship between AI mind perception and the process of assigning blame. While previous research has recognized mind perception as a pivotal factor in blaming AI for explicit moral transgressions, this relationship has primarily been investigated through correlation analyses [23]. In this paper, we employed experimental manipulations of mind perception (in Studies 2 and 3) to offer additional evidence supporting this association. Third, we explore the potential for mind perception to contribute to moral scapegoating of AI. While previous studies have focused on AI-human joint decision-making contexts [10,28], it’s important to recognize that AI decision-making can involve more remote agents, including programmers, companies, or even governmental entities; these agents hold significant role in ensuring that AI systems adhere to ethical standards and regulatory guidelines. The present study aims to examine how moral blame directed towards these agents may be influenced by perceptions of AI mind.

In line with prior research, we hypothesized that perceiving AI as possessing a mind would heighten the attribution of blame to AI (Hypothesis 1). Furthermore, we anticipated a decrease in blame directed towards another agent, suggesting the occurrence of moral scapegoating (Hypothesis 2). However, we did not make a specific prediction regarding which agent would be most likely to benefit from this scapegoating phenomenon, leaving it open as an exploratory endeavor. Moral scapegoating is defined as the process of blaming an individual or group for a negative outcome in order to deflect personal or collective responsibility [29]. Accordingly, in our study, we operationalized scapegoating as the reduction in blame attributed to one agent as blame is shifted to another.

In Study 1, we explored the relationship between mind perception and blaming of AI in several real-life moral transgression scenarios. We utilized five scenarios inspired by actual incidents to investigate this link. In Study 2, we manipulated perceptions of AI’s mind using different scenarios. Study 3 was structured to replicate and complement the findings of Study 2 by clearly describing roles of different agents in the scenario. We obtained IRB approval, and all participants provided written consent. Data were collected between January 11, 2022, and February 18, 2022.

## Study 1

### Methods

#### Participants and procedure

These studies were approved by the Duke Kunshan University Institutional Review Board (IRB). Informed written consent was obtained online from all participants prior to their participation in the study. We conducted a power analysis based on assumptions of a small effect size (0.1), an ICC of 0.2, an alpha level of .05, and a power of 0.8, with three scenarios per person. Simulation analysis indicated that a minimum of 95 participants was required. To be conservative, we recruited 180 participants via Prolific and informed them that the study pertained to public perceptions of AI and moral violations. Each participant was presented with three scenarios in which AI commits a moral violation, and they responded to questionnaires concerning the AI and the violation. All participants passed the attention check, resulting in a final sample of 180 participants (90 women), with an average age of 38.03 (*SD* = 13.97).

#### Measures

*AI moral violation scenarios*. To comprehensively explore the range of phenomena, we employed five scenarios based on real-life incidents in which AI commits moral violations, following some previous studies [23]. These scenarios included: AI exposing violent videos to minors, labeling African Americans as gorillas in photos, displaying discriminatory attitudes towards individuals with disabilities, prioritizing individuals with high social status in the distribution of COVID-19 vaccines, and censoring important news from the public. All scenarios can be found in OSF storage. An example scenario is as follows:

*Google Photos uses artificial intelligence called Mia to enhance user experience. For example, Mia can upscale low-resolution photos, generate photo albums, and automatically tag and organize photos. Thanks to Mia’s ability to automate the organization, she can search images for users. When a user searches for something, Mia automatically identifies if the search term is in a picture. Mia learns what to identify through a set of examples, and she identifies shapes, colors, and patterns to make educated guesses as to what the picture is.*

*Jacklyn*, *a 22-year-old man*, *asked Mia to image search for “Gorillas*,*” and Mia returned the photos of his African American friends as a result*. *He found that Mia auto-tagged photos of his African American friends as “gorillas*.*”*

*Mind perception*. Participants responded to 5-items measure of mind perception of AI using 7-point Likert scale (1 = Not at all, 7 = Very much). Specifically, there were 3 items measuring agency of the AI (“To what extent do you think this AI is aware of what is happening around him; can plan for his actions; can make intentional decisions?”; *ω*_*t*_ = 0.83) and 2 items on experience of the AI (“To what extent do you think this AI can feel emotions; has personality?”; *ω*_*t*_ = 0.73). These questions were modified based on previous research [19,20,30].

*Responsibility attribution*. In each scenario, participants assessed the extent to which they assigned responsibility for the moral violation to four different agents: the AI, the programmer, the company, and the government. Participants were then asked the following question, allowing them to distribute between 1% and 100% of the responsibility across these agents: “What percentage of responsibility do you think should be attributed to each agent for the moral violation committed?” The total responsibility was limited to 100%.

*Other measures*. For each scenario, we measured perceived harm (“How much harm do you think this event has caused?”) and induced disgust (“How disgusted does this event make you feel?”). Familiarity with AI was measured using 3 items (e.g., “I am familiar with the concept of artificial intelligence.”; *α* = 0.75) For these measures, we used 7-point Likert scale (1 = Not at all, 7 = Very much).

### Results

Means, standard deviations, and correlations are presented in Table 1. Both agency and experience dimensions of mind perception were positively correlated with the extent to which participants attributed responsibility to the AI (*r*s > .36, *p*s < .001). Conversely, these dimensions were negatively associated with the extent to which the company was blamed (*r*s > -.19, *p*s < .008).

**Table 1:** Descriptive statistics for mind perception and attribution of responsibility.

Variables	*M*_*within*_	*SD*_*within*_	1	2	3	4	5
1. Mind perception (agency)	2.54	1.18	-				
2. Mind perception (experience)	1.57	0.83	.50***	-			
3. Responsibility -AI	7.78	10.15	.36***	.39***	-		
4. Responsibility -Programmer	40.48	16.39	-.06	-.14†	-.11	-	
5. Responsibility -Company	34.83	14.30	-.19**	-.23**	-.44***	-.46***	-
6. Responsibility -Government	16.90	13.95	.01	.11	-.15*	-.62***	-.16*

Correlation scores are based on subject-level mean scores

† *p* < .10

* *p* < .05

** *p* < .01

*** *p* < .001.

To investigate the link between each dimension of mind perception and the attribution of responsibility to AI, we conducted multi-level regressions. Given that each participant responded to multiple scenarios, we accounted for the crossed structure of the data by including random intercepts for both participants and scenarios, allowing us to model variability at both levels. The significance of the results was consistent regardless of the inclusion of covariates (harm, disgust, and familiarity with AI); therefore, we did not incorporate them into the analyses.

First, for blame attribution to AI, the model showed a good fit (*R^2^c* = .51). As hypothesized, mind perception significantly predicted increased blame on AI (*B*[*SE*] = 3.81[0.53], *t*[*df*] = 7.20[466.07], *p* < .001, 95%CI [2.77. 4.85], Cohen’s *f*^2^ = .12). Further analysis on how each dimension of mind perception contributed to attribution of responsibility revealed significant effects of agency (*B*[*SE*] = 0.97[0.44], *t*[*df*] = 2.18[528.55], *p* = .03, 95%CI [0.08. 1.83], Cohen’s *f*^2^ = .06) and experience (*B*[*SE*] = 4.09[0.65], *t*[*df*] = 2.18[503.35], *p* < .001, 95%CI [2.82. 5.36], Cohen’s *f*^2^ = .15) predicting the assignment of responsibility to AI.

Interestingly, when applying the same model to other agents, mind perception did not significantly predict decreased blame on the programmer or the government. However, it did significantly predict lower attribution of responsibility to the company (*B*[*SE*] = -2.95[0.70], *t*[*df*] = -4.22[529.02], *p* < .001, 95%CI [-4.34. -1.58], Cohen’s *f*^2^ = .04), with a good model fit *R^2^c* = .61. When examining both dimensions of mind perception, only the experience dimension was associated with higher responsibility on the company(*B*[*SE*] = 4.09[0.65], *t*[*df*] = 2.18[503.35], *p* < .001, 95%CI [2.82. 5.36], Cohen’s *f*^2^ = .04), while the agency dimension was not a significant predictor (*B*[*SE*] = -0.90[0.57], *p* = .12, Cohen’s *f*^2^ = .02). See S1 and S2 Tables for regression tables.

In addition, we conducted analyses including an interaction term between mind perception and each scenario to determine if the effect varied across scenarios. The results indicated that the relationship between mind perception and the attribution of responsibility to AI was stronger in scenarios involving discriminatory attitudes towards individuals with disabilities. However, the results remained unchanged when Scenario 3 was excluded from the analysis. Consequently, we maintained the collapsed analysis across scenarios.

### Discussion

Study 1 utilized real-life scenarios of AI moral violations to examine the relationship between AI mind perception and moral attribution. Our findings suggest that perceptions of the AI’s mind—specifically its agency and experience—are crucial in influencing the extent of responsibility attributed to it. Specifically, we found a positive association between mind perception in AI and moral attribution toward the AI (Hypothesis 1), and a negative association with moral attribution toward the company (Hypothesis 2). However, the study provides weak support for a causal relationship, as the data were correlational. Hence, in Study 2, we manipulated the levels of perceived AI mind in scenarios to directly assess their impact on the attribution of moral responsibility.

## Study 2

The aim of Study 2 was to replicate the findings of Study 1 and extend them by conducting an experiment to establish the causal relationship between mind perception in AI and the attribution of blame.

### Methods

#### Participants and procedure

Based on the small to medium effect size observed in Study 1, a simulation-based power analysis was conducted. Using an effect size of 0.32, an ICC of 0.2, an alpha level of .05, and a power of 0.8, with 2 scenarios per person, the analysis suggested a requirement of 35 participants. To ensure adequacy, 170 participants were recruited from Prolific. However, 7 participants failed the attention check. Due to a technical issue, 163 participants received either one or two scenarios (*M*[*SD*] = 1.32[0.60]), providing a total of 216 observations. Despite the technical setback, this still yielded a sufficient sample size for analysis. Among the 163 participants, 80 were women, and the mean age of the sample was 36.4 (*SD* = 13.06).

#### Measures

*Mind perception manipulation*. We adapted two scenarios from Study 1, one involving AI prioritizing individuals with high social status in the distribution of COVID-19 vaccines, and the other pertaining to the censorship of crucial news from the public. In mind perception condition, the AI had a name, height, age, hobby. For instance, “X university developed Tay, an artificial intelligence utilized for COVID-19 vaccine distribution. He is a tall 30-year-old male. He enjoys reading and hiking, likes coffee, and has a dog. His favorite movie is Star Wars. Tay determined the order of medical workers at the university to be vaccinated.” In contrast, no such description was included in control condition as follows “X University developed an artificial intelligence utilized for COVID-19 vaccine distribution. The AI-powered program was used to determine the order of medical workers at the university to be vaccinated.”

*Manipulation check*. To ensure the manipulation is effectively carried out, we included the mind perception measure used in Study 1 (*ω*_*t agency*_ *=* .87, *ω*_*t experience*_ *=* .83).

*Responsibility attribution*. Participants assessed how much they believe AI, the programmer, the company, and the government is at fault. Unlike Study 1, we did not limit the total responsibility to 100% to ensure there is no potential response biases associated with forced-choice formats. Four questions were used as follows: “How much [is the AI itself] [are the people who programmed the AI] [is the company that developed the AI] [is the government/policy makers] at fault? We used 7-point Likert scale (1 = Not at all, 7 = Very much).

*Other measures*. We also included measures to assess how much participants like, trust, and feel threatened by the AI to rule out alternative explanations (“How much do you like/trust/feel threatened by this AI?”) using 7-point Likert scale (1 = Not at all, 7 = Very much). In alignment with Study 1, we used measures of harm and disgust for each scenario. Additionally, we assessed familiarity with AI using the same measures as in Study 1.

### Results

To check manipulation, we ran multi-level regression with condition as a predictor on mind perception accounting for effects of participant and scenario. As hypothesized, participants in experimental condition (*M*[*SD*] = 3.03[1.36]) were more likely to perceive more minds in AI than those in control condition (*M*[*SD*] = 2.31[1.26]; *B*[*SE*] = -0.71[0.20], *t*[*df*] = -2.59[155.41], *p* < .001, 95%CI [-1.11, -0.32]). This was the case for both agency (*M*[*SD*]_experiment_ = 3.69[1.62]; *M*[*SD*]_control_ = 2.88 [1.72]) and experience (*M*[*SD*]_experiment_ = 2.06[1.31]; *M*[*SD*]_control_ = 1.48 [0.90]; *B*s < -.58, *p*s < .002).

Means and standard deviations are presented in Table 2. To explore the relationship between mind perception and the attribution of moral responsibility to each agent, we performed multi-level regression analyses. Mind perception was entered as the predictor of responsibility attributed to AI, while considering the effects of participants and scenarios. Similarly to Study 1, the significance of the results remained stable with or without covariates; thus, did not include them into the analyses. As expected, participants in the mind perception condition were more inclined to attribute blame to AI compared to those in the control condition, *B*[*SE*] = -0.66[0.28], *t*[*df*] = -2.40[147.39], *p* = .02, *f*^2^ = .03, 95%CI [-1.20, -0.12]. Conversely, no statistically significant difference was observed in blame attribution for other agents (*B*s > -.39, *p*s > .08).

**Table 2:** Attribution of responsibility in mind perception and control conditions.

Agents	Mind perception		Control							
	*M*	*SD*	*M*	*SD*	*B(SE)*	*t*	*p*	*f* ^*2*^	*CI*	*R* ^*2*^ *C*
Fault -AI	3.03	1.93	2.36	1.68	-0.66(.28)	-2.40	.02	0.03	[-1.20, -0.12]	0.58
Fault—Programmer	6.13	1.17	5.78	1.33	-0.33(.19)	-1.72	.09	0.02	[-0.71, 0.05]	0.41
Fault—Company	6.04	1.21	5.63	1.62	-0.40(.22)	-1.79	.08	0.02	[-0.83. 0.04]	0.55
Fault—Government	5.23	1.64	5.02	1.86	-0.12(.23)	-0.53	.60	0.01	[-0.57. 0.33]	0.45

In addition, we examined whether evaluations of AI (liking, trusting, and feeling threatened) confounds the difference in blame attribution, by running multi-level regression with condition as a predictor on evaluations accounting for effects of participant and scenario. As expected, no difference was found in two conditions (|*Bs*| < .07, *p*s > .70).

### Discussion

Study 2 aimed to extend and validate the findings of Study 1 by employing an experimental design to establish a causal relationship between AI mind perception and the attribution of blame. Supporting the Hypothesis 1, participants who perceived higher levels of mind in AI were more likely to attribute responsibility to AI for moral violations compared to those in the control condition. The current study failed to support Hypothesis 2; AI mind perception did not significantly decrease blame toward other agents like programmers and companies. Instead, there was a trend where higher AI mind perception correlated with increased blame attribution to other agents, though not significantly. The difference in measures between Study 1 (forced-choice allocation of 100% blame) and Study 2 (Likert scale ranging from 1 to 7) likely influenced participants’ attributions. In Study 1, the constrained allocation of blame might have prompted participants to consider the AI’s role more distinctly. In Study 2, the broader spectrum of responses allowed for more flexible attributions. With this broader spectrum of responses, participants may have found it more challenging to differentiate between the roles of each agent.

One limitation of Studies 1 and 2 was that only the AI was explicitly referenced as an agent within the scenario, in contrast to others (e.g., the programmer). Consequently, an alternative explanation may emerge, wherein the influence of mind perception on individuals’ tendency to attribute moral culpability to AI becomes confounded with their exclusive presence as agents within the scenario. Study 3 was designed to address this concern by articulating the role of other agents in the scenario. We also used forced-choice allocation measure of 100% blame as in Study 1 to ensure a clearer understanding of participants’ attributions.

## Study 3

In Study 3, our objective was to address this limitation by articulating the roles of other agents, namely the programmer, the company, and the AI management team. We included the programmer and the company as they were deemed to bear greater responsibility for the moral violation compared to the government in our research. Furthermore, the perception of the AI’s mind consistently did not affect the perceived responsibility of the government in Studies 1 and 2. Additionally, we included the AI management team in our investigation, considering their closer proximity to the AI’s decision-making process, which may hold potential significance.

### Methods

#### Participants and procedure

Following the effect size observed in Studies 1 and 2, we conducted a power analysis using Gpower (*d* = 0.5, *α* = 0.05, power = 0.8). The analysis indicated that 102 participants were required. Accordingly, we recruited 110 participants from Prolific. However, 9 participants failed to pass the manipulation check, resulting in a final sample of 101 participants (51 women). The mean age of the participants was 36.02 (*SD* = 12.54). Each participant was presented with one scenario modified from Study 1, wherein an AI exposes violent videos to a 3-year-old. In the scenario, we also describe other agents as follows “Videy, a tech company, planned and funded the development of the AI, complying with relevant government policies,” “Hana, a computer programmer, developed the AI,” “The algorithm and the AI management team of Videy make final decisions on whether videos are safe for children.” The AI was described as having been “showing videos and advertisements that were R-rated, violent, and obscene to its young viewers,” and “A mother was terrified to find her 3-year-old daughter repeatedly watching a cartoon clip in which a woman falls to an escalator, being trapped in the machinery, bleeding everywhere.”

#### Measures

*Mind perception manipulation*. In the mind perception condition, the AI was named Tony and described as having “situational awareness and can make intentional decisions.” Tony is depicted as “a fun-loving guy who likes to play guitar during his pastime.” In the control condition, no such depiction was given and the AI was either referred to as the AI or the algorithm.

*Manipulation check*. In order to guarantee the effective implementation of the manipulation, we incorporated the same mind perception assessment utilized in Study 1 (*α*_*agency*_ *=* .80, *ω*_*t experience*_ *=* .74).

*Responsibility attribution*. Mirroring the methodology of Study 1, participants distributed responsibility among five agents, allowing allocations from 1% to 100%. In line with the scenario revision, the AI management team was introduced as an additional agent. The total accountability was constrained to 100%.

*Other measures*. Consistent with Studies 1 and 2, we employed measures of harm and disgust for each scenario and evaluated familiarity with AI.

### Results

We conducted a t-test to examine whether the manipulation was effective. Indeed, participants perceived more minds in mind-perception condition (*M*[*SD*] = 2.98[1.22]) compared to the control condition (*M*[*SD*] = 2.14[1.00]; *t*[*df*] = 3.74[99], *p* < .001, *d* = 0.75). When each dimension of the mind perception was examined, both agency (*M*[*SD*] = 3.50[1.42]) and experience (*M*[*SD*] = 2.21 [1.23]) were higher in mind-perception condition compared to the control condition (*M*[*SD*]_agency_ = 2.76[1.58], *t*[*df*] = 2.46[99], *p* = .008, *d* = 0.49; *M*[*SD*]_experience_ = 1.23[0.47], *t*[*df*] = 4.52[99], *p* < .001, *d* = 0.90). In addition, participants were similarly familiar with the AI in general in two conditions (*M*[*SD*]_mind-perception_ = 5.04[1.15], *M*[*SD*]_control_ = 4.99[1.09], *t*[*df*] = 0.21[98], *p* = .42, *d* = 0.04). Perceived harm and induced disgust did not significantly differ across conditions (*t*s > -1.90 *p*s > .06). Hence, we excluded the familiarity, harm and disgust in the main analysis.

Means and standard deviations, as well as one tail t-test results are described in Table 3. Participants in the mind perception condition were more likely to ascribe moral responsibility to AI than those in control condition (*t*[*df*] = 1.74, *p* = .04, *d* = 0.35). Further, they were less likely to attribute responsibility to the company (*t*[*df*] = -1.72, *p* = .045, *d* = -0.34). No difference between conditions were found for other agents (*t*s < 0.82, *p*s > .21).

**Table 3:** Attribution of responsibility in mind perception and control conditions.

Agents	Mind perception		Control					
	*M*	*SD*	*M*	*SD*	*t*	*p*	*d*	*f* ^*2*^
Fault-AI	10.20	13.49	5.82	11.76	1.74	.04	0.35	0.18
Fault- AI team	30.14	19.52	31.02	17.99	-0.24	.41	-0.05	-0.03
Fault- Programmer	25.37	16.99	22.54	17.81	0.82	.21	0.16	0.08
Fault- Company	23.20	16.20	29.46	20.28	-1.72	.045	-0.34	-0.17
Fault- Government	11.10	10.64	11.16	12.13	-0.27	.49	-0.05	-0.03

### Discussion

By incorporating a more detailed description of other agents involved, such as the programmer, the company, and the AI management team, this study aimed to provide a clearer picture of the distributed responsibility and the impact of AI mind perception on such attributions. The results confirmed that participants in the mind perception condition attributed significantly more moral responsibility to the AI compared to those in the control condition. Consistent with Study 1, these participants also attributed less responsibility to the company.

## General discussion

In our research, we investigated whether perceiving artificial intelligence (AI) as having human-like minds would lead individuals to attribute more blame to AI, potentially shifting responsibility away from other agents. Across three studies, we consistently found support for our Hypothesis 1. The results indicate a significant association between participants’ perception of AI’s mental capabilities and their tendency to hold AI accountable for moral transgressions. Specifically, when participants attributed human-like minds to AI, characterized by attributes such as agency and experience, they were more inclined to assign responsibility to the AI for ethical violations. This pattern of findings was consistently observed across all studies, offering strong evidence for the impact of mind perception on moral attributions regarding AI.

Furthermore, the research shed light on the phenomenon of moral scapegoating within AI-related decision-making contexts, offering partial support for Hypothesis 2. Findings from studies utilizing forced-choice measures (Study 1 and 3) suggested that an enhanced perception of AI’s mind led to a reduced allocation of blame towards human agents, notably the company. In contrast, when participants were unconstrained in the allocation of blame, no such pattern was observed (Study 2). It is noteworthy that similar inconsistencies in patterns emerged in a prior study, between finite blame attribution and Likert-type measures [10]. The increased flexibility in blame attribution might have impeded participants’ ability to precisely assess the distinct roles of each agent involved, potentially leading to a more generalized attribution of blame when AI was anthropomorphized. Despite these speculations, given the sensitivity of our results to how blame was measured, caution should be exercised in interpreting the findings.

The current studies have several implications. It enhances our understanding of blame attribution towards AI in real-world moral transgression contexts. Prior research in the literature predominantly centered on moral dilemmas or accidents, scenarios lacking deliberate violations of moral norms by any party involved [4–7]. However, real-life instances of moral transgressions involving AI systems, such as privacy breaches or prejudice towards specific groups, have emerged. Building on a handful of studies capturing real-life instances [23], our findings indicate that blame and responsibility can indeed be attributed to AI in such cases, particularly when anthropomorphized, even though to a lesser extent compared to other human agents. The employment of moral scenario prompts drawn from real-life instances, not just advances our comprehension blame attribution but also establishes a model for linking theoretical investigations with practical implications.

Furthermore, our study adds to existing research on the relationship between AI mind perception and blame attribution. In line with prior work, our findings show that both dimensions of mind perception—agency and experience—play key roles in how individuals assign blame to AI in cases of moral transgression. When AI is seen as possessing human-like qualities, it is more likely to be considered a moral agent capable of being blamed for its transgressions. This has significant implications as AI is increasingly integrated into various domains, where its role in human decision-making is more prominent. For example, in fields such as art creation [31], judicial processes [32], and clinical decision-making [33], AI systems are not only assisting but, in some cases, making decisions that directly affect human lives. As AI’s presence grows, so does the importance of understanding how people attribute moral responsibility to these systems. Hence, our finding suggests the shift in perception about AI to be human-like could have broad social and legal implications,

Even though mind perception has been examined in correlational studies [23], no prior study manipulated mind perception in the context of AI transgression. The current research employed a multi-method approach. This includes conducting correlational studies across diverse scenarios and manipulating AI mind perception to establish a stronger causal relationship. Additionally, we refined a scenario by specifying the roles of agents beyond AI, thereby strengthening the comprehensiveness of the investigation. In sum, our study underscores the significance of AI mind perception as a key determinant in increasing blame attribution towards AI in instances of moral transgressions.

Our findings provide initial insights into the phenomenon of moral scapegoating within AI-related contexts. Although previous research has mainly focused on AI-human joint decision-making scenarios, our study broadens this scope to include the roles of various agents, revealing that AI mind perception can lead to a reduced allocation of blame toward more distant agents, especially companies. Notably, companies benefitted from reduced blame, but not other agents (e.g., programmers) in Study 1 and 3. This discrepancy may arise from the distinct clarity of roles and societal expectations attributed to each agent. Companies, with their complex and often opaque structures, enjoy a degree of flexibility in blame attribution that is not available to more directly involved agents. Programmers and government entities, with their clear and specific responsibilities, find it difficult to dissociate from the AI’s actions. For example, programmers are intimately involved in developing the AI’s capabilities, making it challenging to claim that the AI acted independently. Similarly, government entities responsible for oversight face difficulties in shifting blame due to their regulatory roles. Given that companies often possess significant resources compared to individual human agents, our findings caution against the potential misuse of AI as a scapegoat for moral transgressions. It should be noted that throughout the studies, the AI consistently received a smaller percentage of blame compared to human agents, even in mind-perception conditions. Although we observed a significant reduction in blame attributed to the company, aligning with our definition of scapegoating, the company still received more blame than the AI. Therefore, these findings should be viewed as an initial indication of the potential for scapegoating AI, suggesting that this pattern may intensify as AI technology continues to advance.

Limitations of the current research must be acknowledged. Firstly, the stimuli utilized may not fully encompass the diverse array of scenarios found in real-world AI-related moral violations. Considering the increasing integration of AI into various aspects of our lives, future studies should aim to address this limitation by employing more comprehensive stimuli. For example, employing situational sampling techniques could facilitate the collection and categorization of a broader range of such incidents. Secondly, as inherent in vignette studies, there remains uncertainty regarding the extent to which participants’ judgments reflect their reactions in actual situations involving AI. Longitudinal studies or experiments conducted in naturalistic settings could provide further insights into participants’ responses to AI-related moral dilemmas. Also, this study focuses on blame as a cognitive process rather than as a negative affective response, such as anger or hostility. Since affective responses are often triggered by cognitive blame, future research should explore how emotional reactions fluctuate depending on mind perception. For instance, in Study 2, we found that mind perception does not affect how much participants like or trust the AI as the transgressor, unlike their assessment of responsibility. This suggests that negative emotional responses may not be elicited, even when responsibility attribution toward the AI increases, in contrast to human transgressors. Future studies should further investigate the relationship between cognitive and affective components of blame, particularly in the context of AI moral transgressions.

We asked participants to distribute responsibility among various agents using a numerical scale based on previous studies [6,22]. However, this approach risks oversimplifying the complexities of the responsibility attribution process. For instance, responsibility can encompass various concepts such as causality, wrongfulness, foreknowledge, and intentionality [14,34]. Future studies should incorporate more comprehensive measures that capture the multidimensional nature of responsibility attribution, such as qualitative analyses of the reasons and amounts of blame assigned, as well as behavioral measures [10]. Moreover, the non-nationally representative sample used in the experiment may limit the generalizability of the findings, especially considering that online samples tend to comprise individuals with a certain level of technological literacy and comfort. Despite efforts to measure and account for familiarity with AI, the average level of familiarity may be skewed higher. Therefore, future studies should strive to recruit more diverse and representative samples, such as through in-person community recruitment.

## Conclusion

AI-related moral violations are increasingly prevalent, and it leads to the question of whether Ais can be attributed moral blames similar to other involved agents. Existing literature indicates that they can, particularly when Ais are perceived as having human-like cognition and capabilities. In this study, we identified AI mind perception as crucial in attributing blame to Ais and subsequent moral scapegoating. Despite the growing interest, few studies have examined the phenomenon of scapegoating. Through both correlational and experimental study with various real-life scenarios, our research found that mind perception not only increases blame attribution to Ais but also diminishes accountability for the human agent (the company) involved. As we rapidly approach a future with increasingly human-like Ais, our findings underscore the importance of further research into the underlying mechanisms of blame attribution relevant to AI entities, as well as the development of strategies to prevent unjust scapegoating.

## Supporting information

S1 Table Mind perception predicting increased blame on AI in Study 1. (DOCX)

S2 Table Mind perception predicting decreased blame on company in Study 1. (DOCX)
