# Supplementary material for: It’s the AI’s fault, not mine: Mind perception increases blame attribution to AI
Source: PLoS One. 2024 Dec 18;19(12):e0314559. doi: 10.1371/journal.pone.0314559 (PMC11654982; doi:10.1371/journal.pone.0314559)
Supplement: S1 Table — (DOCX) [file pone.0314559.s001.docx]

| Predictor Variable | *B* | *SE* | *t* | *df* | *p* | 95% CI |
| --- | --- | --- | --- | --- | --- | --- |
| Intercept | -1.10 | 1.40 | -0.79 | 87.95 | .43 | [-3.83, 1.63] |
| Agency | 0.97 | 0.44 | 2.18 | 528.55 | .03 | [0.08, 1.83] |
| Experience | 4.09 | 0.65 | 6.31 | 503.35 | < .001 | [2.82, 5.36] |
| R² (conditional) |  |  |  |  |  | .51 |

S1 Table.

Mind perception predicting increased blame on AI in Study 1.
