# Supplementary material for: It’s the AI’s fault, not mine: Mind perception increases blame attribution to AI
Source: PLoS One. 2024 Dec 18;19(12):e0314559. doi: 10.1371/journal.pone.0314559 (PMC11654982; doi:10.1371/journal.pone.0314559)
Supplement: S2 Table — (DOCX) [file pone.0314559.s002.docx]

S2 Table.

Mind perception predicting decreased blame on company in Study 1.

| Predictor Variable | *B* | *SE* | *t* | *df* | *p* | 95% CI |
| --- | --- | --- | --- | --- | --- | --- |
| Intercept | 41.75 | 2.08 | 20.07 | 48.41 | < .001 | [37.65, 45.80] |
| Agency | -0.90 | 0.57 | -1.57 | 521.93 | .12 | [-2.04, 0.22] |
| Experience | 4.09 | 0.65 | 2.18 | 503.35 | < .001 | [2.82, 5.36] |
| R² (conditional) |  |  |  |  |  | .61 |
